# Guanfacine as an effective drug for the treatment of tic disorder. Case report

**DOI:** 10.1192/j.eurpsy.2021.1673

**Published:** 2021-08-13

**Authors:** P. Del Sol Calderon, A. Izquierdo, M. García Moreno

**Affiliations:** Psychiatry, Hospital Universitario Puerta de Hierro, Majadahonda, Spain

**Keywords:** Tics, Treatment, child and adolescent

## Abstract

**Introduction:**

A 15-year-old man who comes to the consultation referred from neurology for a poorly progressive tic disorder that is resistant to treatment. This is a patient with no relevant medical history. He has good academic performance, without symptoms of inattention, hyperactivity or impulsivity. He does not present buccophonatory tics

**Objectives:**

To Show guanfacine as a efficient treatment fot tics disorder

**Methods:**

Case report

**Results:**

He has presented complex motor tics as cervical contractions that have had to receive physiotherapeutic treatment. He also presents simple ocular tics. The patient at our evaluation is on a 4 mg dose of pimozide without response. He was previously on risperidone. It was decided to start treatment with guanfacine up to 5 mg with reduction of pimozide, to 1 mg. The patient with this dose adjustment shows a notable improvement in the frequency and intensity of the tics, both cervical and ocular. The patient also refers to a feeling of being calmer and being able to face stressful situations such as being exposed to social relationships, intervening more in class without increasing their anxiety levels.

**Conclusions:**

Guanfacine is a selective alpha-2a adrenergic receptor agonist that has an indication for attention deficit hyperactivity disorder. Like its precursor, clonidine, there is more and more literature that proposes this medication and others for ADHD as useful drugs in pathologies such as tic disorder. It has a safe use profile, which with progressive adjustment and hardly any side effects is placed as a treatment to take into account in this pathology.

**Disclosure:**

No significant relationships.

